# Fitting data reveals the complexities of NADP as a Ca^2+^ ATPase inhibitor

**DOI:** 10.14814/phy2.14259

**Published:** 2019-10-09

**Authors:** Agustin Guerrero‐Hernandez

**Affiliations:** ^1^ Biochemistry Department Cinvestav Mexico City Mexico

**Figure 1:**
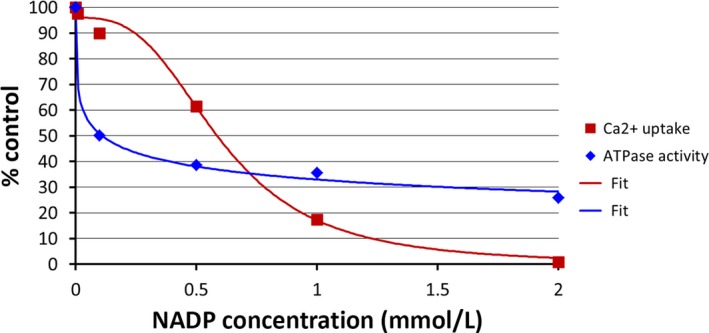
Fitting data extracted from figures 2 and 10 of Wang et al. The microsomal Ca^2+^ uptake activities at different NADP concentrations were taken from Figure 2 and they are shown as red squares. The fitting of these data (red line) was achieved using a single inverse saturation curve with an IC_50_ = 0.6 mmol/L and a Hill coefficient, *n* = 3.0 with an *R* ^2^ = 0.994. The Ca^2+^/Mg^2+^ ATPase activities at different NADP concentrations were taken from Figure 10 and shown as blue diamonds. The fitting of these data (blue line) with a single inverse saturation curve resulted in an IC_50_ = 0.11 mmol/L and a Hill coefficient, *n* = 0.32 with an *R* ^2^ = 0.996.

**Figure 2:**
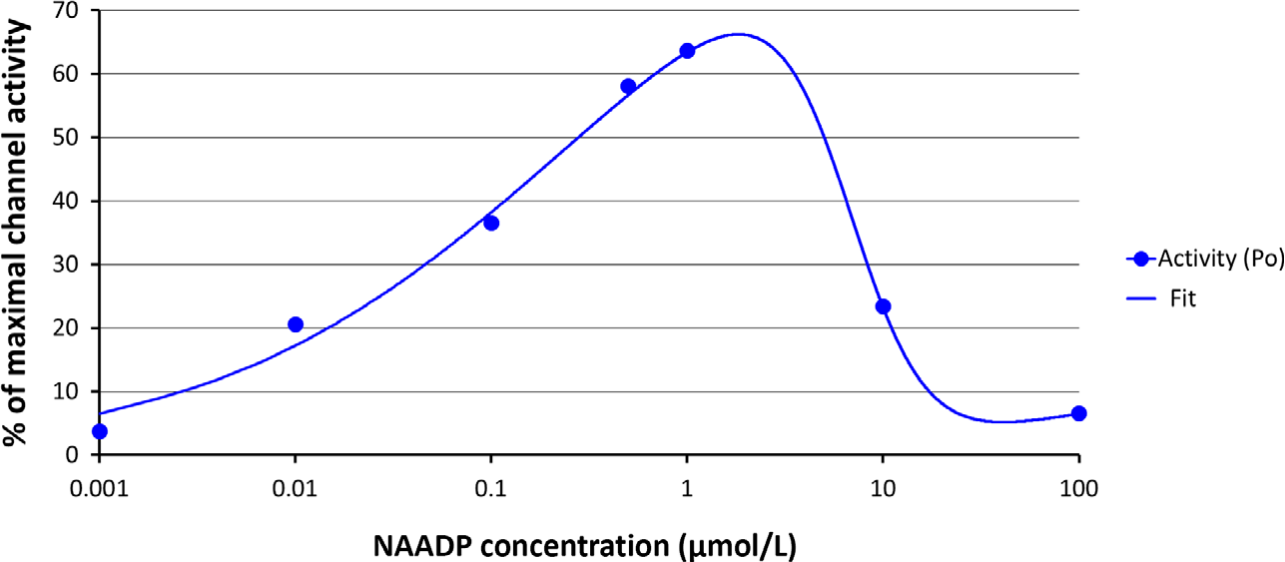
NAADP‐induced ion channel activity data extracted from Figure 3 in Zhang and Li (2007) are shown as blue circles. The fitting of the data (blue line) was obtained with the difference between two saturation curves where the maximal activation was considered to be 100% activity and the maximal inactivation was 88% of maximal activity. The activation curve has an EC_50_ = 0.27* μ*mol/L and a Hill coefficient, *n* = 0.472 while the inactivation function has an EC_50_ = 6.8 *μ*mol/L and *n* = 2.17 with an *R* ^2^ = 0.993. Due to the presence of these two opposing effects, the maximal ion channel activity induced by NAADP is not higher than 70% and the inactivation by NAADP is not complete.

Wang et al. (2019) propose that NADP inhibits SERCA pump and that this explains the inhibitory effect of NADP on Ca^2+^ uptake by rat liver microsomes. The analysis of these data extracted from figures 2 and 10 is shown in Figure 1, the data on Ca^2+^ uptake from Figure 2 by Wang et al. are shown as red squares and data on Ca^2+^/Mg^2+^ ATPase activity from figure 10 are shown as blue diamonds. The fitting of these data using one single inverse saturation function ($100*{\text{IC}}_{50}^{n}/\left({\text{IC}}_{50}^{n}+{\left[\text{NADP}\right]}^{n}\right)$) with only two parameters (IC_50_ and *n*, Hill coefficient) is shown in Figure 1. It is evident that the inhibitory effect of NADP on microsomal ATPase activity (blue line) has a different concentration dependence that the effect of NADP on the accumulation of Ca^2+^ by rat liver microsomes (red line). The former has an IC_50_ of 0.11 mmol/L and a small Hill coefficient (0.32) while the latter has an IC_50_ of 0.6 mmol/L and a Hill coefficient of 3.0 (Fig. 1). The authors argue that SERCA pump accounts for only 14% of the total ATPase activity (Wang et al. 2019). However, this does not seem to explain that the effect of NADP on ATPase activity and microsomal Ca^2+^ uptake occurs in a different concentration range resulting from different Hill coefficients. NAADP was discovered as a Ca^2+^ releasing agent that came as a contaminant in NADP (Lee and Aarhus 1995). NAADP not only is highly potent in releasing Ca^2+^ from microsomes derived from different tissues, but also from rat liver microsomes that were Ca^2+^ loaded by the activity of SERCA pump (Mándi et al. 2006). NAADP also induces a substantial inactivation of its Ca^2+^ releasing activity (Lee 1997), this situation has also been shown in NAADP‐responding channels (Fig. 2) isolated from liver lysosomes (Zhang and Li 2007) or for Ca^2+^ released from rat liver microsomes (Mándi et al. 2006). Wang et al. did experiments to discard the participation of NAADP. However, to achieve this goal it is essential to consider that NAADP induces a time‐ and concentration‐dependent self‐inactivation (Lee 1997; Mándi et al. 2006). It is clear then that there is an optimal concentration of NAADP to induce Ca^2+^ release from microsomes that is near 1 *μ*mol/L and that this inactivation is not complete (Fig. 2).

Nevertheless, Wang et al. used NAADP at 1 mmol/L, a concentration that induces a significant inactivation of NAADP‐triggered Ca^2+^ release activity. Accordingly, they saw that this high concentration of NAADP decreased Ca^2+^ uptake and more importantly, fully inhibited the effect of NADP (figure 5 of Wang et al.). This result is compatible with the idea that the effect of NADP depends on the activity of NAADP. Figure 6 of Wang et al. shows that NADP is not increasing Ca^2+^ efflux from microsomes, but the conditions used here are different to those used to assess Ca^2+^ uptake, for instance, the presence of oxalate, ATP, and a higher concentration of Mg^2+^ in the latter compared with the former. So it could be that NAADP activity is different in these two different assay conditions. Finally, the use of trans‐Ned‐19 to discard participation of NAADP resulted in unexpected observations. All the inhibitors tested (2‐APB, ruthenium red, and dantrolene) increased the inhibitory effect of NADP on Ca^2+^ uptake, except for trans‐Ned‐19. There is no clear explanation for these results. In any event, the data shown in table 1 of Wang et al. is not strong enough to discard the participation of NAADP that might be present in their NADP solution. In conclusion, the work by Wang et al. has not entirely ruled out the possibility that traces of NAADP in their NADP solution are responsible for activating a Ca^2+^ release channel resulting in the apparent NADP inhibitory effect on Ca^2+^ uptake by liver microsomes.
